# Outcomes of early versus delayed weight-bearing with intramedullary nailing of tibial shaft fractures: a systematic review and meta-analysis

**DOI:** 10.1007/s00068-022-01919-w

**Published:** 2022-03-03

**Authors:** Ameya Bhanushali, Joshua G. Kovoor, Brandon Stretton, James T. Kieu, Rebecca A. Bright, Joseph N. Hewitt, Christopher D. Ovenden, Aashray K. Gupta, Mohamed Z. Afzal, Suzanne Edwards, Ruurd L. Jaarsma, Christy Graff

**Affiliations:** 1 Department of Orthopaedics and Trauma, Royal Adelaide Hospital, Adelaide, South Australia Australia; 2 Discipline of Surgery, University of Adelaide, The Queen Elizabeth Hospital, 28 Woodville Road, Woodville, SA 5011 Australia; 3 University of Adelaide, Adelaide, South Australia Australia; 4 Discipline of Surgery, University of Adelaide, Women’s and Children’s Hospital, Adelaide, South Australia Australia; 5 Discipline of Surgery, University of Adelaide, Royal Adelaide Hospital, Adelaide, South Australia Australia; 6 Department of Surgery, Lyell McEwin Hospital, Adelaide, South Australia Australia; 7 School of Public Health, Adelaide Health Technology Assessment, University of Adelaide, Adelaide, South Australia Australia; 8 Flinders Medical Centre, Department of Orthopaedics and Trauma, Adelaide, South Australia Australia; 9 College of Medicine and Public Health, Flinders University, Adelaide, South Australia Australia

**Keywords:** Weight-bearing, Tibial shaft, Intramedullary nail, Fracture union, Early weight-bearing, Trauma

## Abstract

**Purpose:**

Early weight bearing (EWB) is often recommended after intramedullary nailing of tibial shaft fractures, however, the risks and benefits have not been critically evaluated in a systematic review or meta-analysis. Therefore, the aims of this study were to perform a systematic review and meta-analysis comparing EWB and delayed weight-bearing (DWB) after intramedullary nailing of tibial shaft fractures and assess the relationship between weight-bearing, fracture union and healing.

**Method:**

This review included studies comparing the effects of EWB, defined as weight-bearing before 6 weeks, and DWB on fracture union and healing. PubMed, Embase, CINAHL, and the Cochrane Library were searched from inception to 9 May 2021. Risk of bias was assessed using the Down’s and Black Checklist and Cochrane Risk of Bias Tool 2.0. Data were synthesised in a meta-analysis, as well as narrative and tabular synthesis.

**Results:**

Eight studies were included for data extraction and meta-analysis. The analysis produced mixed results and found a significant decrease in mean union time (−2.41 weeks, 95% confidence interval: −4.77, −0.05) with EWB and a significant Odd’s Ratio (OR) for complications with DWB (OR: 2.93, 95% CI: 1.40, 6.16). There was no significant difference in rates of delayed union, non-union, re-operation and malunion.

**Conclusion:**

The included studies were of moderate risk of bias and demonstrated shorter union time and fewer complications with EWB. However, current evidence is minimal and has significant limitations. The role of EWB in high-risk patients is yet to be examined. Further well-designed, randomised studies are required on the topic.

**Supplementary Information:**

The online version contains supplementary material available at 10.1007/s00068-022-01919-w.

## Introduction

Tibial shaft fractures are the most common long-bone fracture, accounting for 1.9% of all fractures [1, 2]. Intramedullary nailing (IMN) is the gold standard for treatment of displaced tibial shaft fractures in adults [3–6]. Initially, most of the weight-bearing load passes through the nail, and as the fracture heals, the load is gradually transferred to the bone [4]. This process makes early weight-bearing (EWB), defined as within 6 weeks after surgery [7, 8], possible.

The scientific basis for the potential benefits of EWB is well known. Weight bearing facilitates mechanical loading of the bone and subsequently increases bone deposition via mechanotransduction [9]. EWB may accelerate this process and increase the amount of early callus formation [10]. Furthermore, non-weight-bearing even for brief periods leads to significant bone loss in the tibia, which may take up to 18 months to recover [11]. A similar process affects skeletal muscle, causing significant atrophy after as little as 10 days of immobility [12, 13]. Weight bearing may also reduce post-operative ankle stiffness [14]. Accordingly, it has been hypothesised that EWB may lead to higher fracture union rates, earlier fracture union, decreased muscle atrophy and ankle stiffness, and earlier return to work [8, 15].

However, re-operation is already required in 14% of patients after tibial IMN, with delayed union, non-union, malunion, infection and implant failure being the most common causes [3]. With the increased mechanical stress on the healing bone that EWB confers, the possibility for increases in complications such as malunion, implant failure and re-operation must be considered before it can be recommended. For this reason, DWB is commonly prescribed for unstable fractures, patients with bone loss and osteoporotic patients, and the role of EWB in these groups has not been determined. To inform post-operative care, we aimed to perform a systematic review and meta-analysis comparing EWB and DWB after IMN of tibial shaft fractures and determine effects on postoperative fracture union and healing. There are currently no universal guidelines for weight-bearing after tibial IMN [8].

## Methods

This review was registered with the International Prospective Register of Systematic Reviews (PROSPERO, CD42021255704) prior to commencement and was reported in accordance with the Preferred Reporting Items for Systematic Reviews and Meta‐Analyses (PRISMA) and Meta-analysis Of Observational Studies in Epidemiology (MOOSE) guidelines [16, 17].

### Search strategy and selection criteria

PubMed (incorporating MEDLINE), Embase, CINAHL, and the Cochrane Library were searched from inception to 9 May 2021. The search strategy incorporated the following terms: “weight*” AND “tibia*” AND “fractur*” AND “nail*”. Searches were supplemented by consultation of current contents, reviews, and original research relating to weight-bearing after IMN of tibial shaft fractures identified through targeted searches of Google Scholar and PubMed.

Inclusion criteria were set to include studies comparing outcomes of EWB versus DWB after IMN in tibial shaft fractures. EWB was defined as permission to bear full bodyweight prior to 6 weeks post-operatively [7, 8], whilst DWB was defined as permission to bear full bodyweight at 6 weeks or later. EWB was further subcategorised into immediate weight-bearing (IWB), weight-bearing before 2 weeks, between 2 and 4 weeks and between 4 and 6 weeks. IWB was defined as permission to bear full bodyweight from day one post-operatively. Outcomes of interest included time to fracture union, non-union rate, malunion rate, re-operation rate, pain and total complications. Fracture union was defined by the bridging of at least three of four cortices on two orthogonal radiographs.

Experimental, quasi-experimental and observational study designs were considered. Case series, case reports and opinion articles were excluded. Searches were not restricted by study setting or language and all studies from database inception to 9 May 2021 were considered.

### Data extraction

Studies were reviewed for inclusion by two independent investigators (A.B. and J.T.K.). Studies were screened for inclusion by title and abstract and then full text using a web application (Rayyan, Qatar Computing Research Institute, Ar-Rayyan, Qatar). Discrepancies were resolved by consensus. Data were extracted by two independent investigators (A.B and J.T.K.) and discrepancies were resolved by a third investigator (C.D.O.). Extracted data included research design, study setting, population characteristics, intervention characteristics, comparator characteristics, timeframe for follow-up, quantitative and qualitative outcomes, source of funding and reported conflicts of interest, methodological quality information, and other information relevant to the review questions. Data were synthesised in narrative and tabular formats.

### Data analysis

Data analyses were performed using Stata Statistical Software: Release 15.1 College Station, TX: StataCorp LP. In this meta-analysis, for one continuous variable (union time), mean differences with 95% confidence intervals (CIs) were calculated, and for five dichotomous variables (delayed union, non-union, malunion, re-operation and complications), odds ratios (OR) and 95% CIs were calculated, for each study and then for all the studies combined. A variable was included in the meta-analysis if at least two of the eight journal articles involved had sufficient values for that variable. Subgroup meta-analysis was not possible as studies did not report outcomes for EWB and DWB by fracture type.

The *I*^2^ statistic was used to evaluate heterogeneity (with *I*^2^ > 50% indicating significant heterogeneity) as was Cochran’s Q *P* value (with *P* value < 0.05 indicating significant heterogeneity). A random effects model was used throughout. A *P* value of < 0.05 denoted statistical significance. A Funnel plot was created for each variable to test for publication bias. An Egger’s Test has been performed for each variable to test for small-study effects.

Two independent reviewers (B.S and R.A.B) assessed risk of bias and methodological quality in the included studies. For this, the Cochrane Risk of Bias Tool 2.0 [18] was used to evaluate randomised controlled trials (RCTs) and the Downs and Black Checklist was used to evaluate non-randomised observational studies (NSRIs) [19].

## Results

Searches identified a total of 2019 records (1478 unique reports), from which 19 full-text articles were retrieved and 8 of these studies were included in the systematic review (Appendix 3). A list of studies excluded at full-text review, with justification of exclusion for each potentially relevant study, can be found in Appendix 3. The characteristics of the included studies are outlined in Appendix 5. Included studies consisted of one RCT [8], two prospective cohort studies [20, 21], one retrospective case–control study [6] and four retrospective cohort studies [7, 15, 22, 23].

### Union time

The mean union time and standard deviation of union time for EWB and DWB groups were pooled across four studies using a random effects model [6–8, 23]. Heterogeneity in the study estimates was assessed using the *I*^2^ statistic (0.0%) and Cochran’s Q *P* value (0.437) which showed no heterogeneity. EWB had an overall mean difference in union time across the studies of −2.41 (95% CI: −4.77, −0.05), equating to a mean union time in the EWB group that was 2.41 weeks less than mean union time in the DWB group (Appendix 6). This was a statistically significant result as zero was not within the 95% CI. A Funnel plot showed no publication bias (Appendix 7) and Egger’s Test showed no small-study effects (*P* value = 0.995) (Appendix 8).

### Delayed union

Log Odds Ratio (OR) was calculated for the percentage of delayed unions for EWB and DWB groups, as well as the standard error of the log OR. The log ORs were pooled across four studies using a random effects meta-analysis model [6–8, 21]. Heterogeneity in the study estimates was assessed using the *I*^2^ statistic (48.7%) and Cochran’s Q *P* value = 0.119 which show no heterogeneity. The OR of delayed unions across the studies was 1.44 (95% CI: 0.52, 4.01) equating to odd of delayed unions of 1.44 times that of the EWB group (Appendix 9). However, this result was not statistically significant. A Funnel plot showed no publication bias (Appendix 10) and Egger’s Test indicated no small-study effect (*P* value = 0.138) (Appendix 11).

### Non-union

Log ORs were calculated for non-union rates for the EWB and DWB groups, as well as the standard error of the log OR. The log ORs were pooled across six studies using a random effects model [6–8, 21–23]. Heterogeneity in the study estimates was assessed using the *I*^2^ statistic (0%) and Cochran’s Q *P* value = 0.494 which show no heterogeneity. The OR of non-unions across the studies is 1.59 (95% CI: 0.72, 3.50), equating to odds of having a non-union in the DWB group 1.59 times that of the EWB group (Appendix 12). However, this result was not statistically significant. A Funnel plot showed no publication bias (Appendix 13) and Egger’s Test indicates no small-study effect (*P* value = 0.965) (Appendix 14).

### Malunion

Log OR was calculated for the percentage of malunions for each group, as well as the standard error of the log OR. The log ORs were pooled across two studies using a random effects meta-analysis model [6, 8]. Heterogeneity in the study estimates was assessed using the *I*^2^ statistic (0%) and Cochran’s Q *P* value = 0.733 which show no heterogeneity. The OR of malunions across the studies has a very wide confidence interval, likely due to multiple zero values for malunion, and accordingly overall ORs could not be calculated (Appendix 15). A Funnel plot showed no publication bias (Appendix 16) and Egger’s Test did not converge (Appendix 17).

### Re-operation

Log OR was calculated for the percentage of reoperations for EWB and DWB groups, as well as the standard error of the log OR. The log ORs were pooled across two studies using a random effects meta-analysis model [6, 7]. Heterogeneity in the study estimates was assessed using the *I*^2^ statistic (0%) and Cochran’s Q *P* value = 0.649 which show no heterogeneity. The OR of reoperations across the studies is 2.48 (95% CI: 0.77, 7.92) in DWB, equating to the odds of having a re-operation in the DWB group 2.48 times that of the EWB group (Appendix 18). However, this result was not statistically significant. A Funnel plot showed no publication bias (Appendix 19) and Egger’s Test does not converge (Appendix 20).

### Complications

Log OR was calculated for the percentage of complications for EWB and DWB groups, as well as the standard error of the log OR. The log ORs were pooled across two studies using a random effects meta-analysis model [6, 22]. Heterogeneity in the study estimates was assessed using the *I*^2^ statistic (0%) and Cochran’s Q *P* value = 0.931 which show no heterogeneity. The OR of complication across the studies is 2.93 (95% CI: 1.40, 6.16) with DWB, meaning the odds of having a complication in the DWB group were 2.93 times that of the EWB group (Appendix 21) This result was statistically significant. A Funnel plot shows no publication bias (Appendix 22) and Egger’s Test does not converge (Appendix 23).

### Risk of bias

The included non-randomised studies were of moderate methodological quality upon using the Downs and Black checklist [19]. Overall percentages on risk of bias assessments for the included studies can be found in Appendix 5 and the individual breakdown of these scores can be found in Appendix 24. Mean scores representing the average of the two reviewer scores, were calculated for each question. Calculated means were as follows: total mean score 19 out of 32 (range 18–20); reporting sub-scale mean score 9.4 out of 11 (range 9–10); external validity sub-scale mean score 2.1 out of 3 (range 1–3); bias sub-scale mean 4.9 out of 7 (range 4–5); confounding sub-scale mean 2.6 out of 6 (range 2–3); power sub-scale mean 0 out of 5 (range 0).

The included RCT was of good methodological quality upon using the Cochrane Risk of Bias Tool 2.0 [18]. The overall risk of bias and risk of bias in all five domains was scored as low by both reviewers (Appendix 25).

## Discussion

This systematic review and meta-analysis investigated studies of moderate quality comparing outcomes of EWB with DWB after IMN of tibial shaft fractures. This review indicates there are benefits associated with EWB including earlier union time and lower rates of overall complications. There were no significant differences in rates of delayed union, non-union, malunion or re-operation, although more cases of non-union, malunion and re-operation were recorded in the DWB group. One study reported no significant differences in Short Musculoskeletal Function Assessment (SMFA) [24] domains [8], one reported equivocal anterior knee pain, knee and ankle stiffness between groups [6], and one reported equivocal mobility at a year post-operatively [21].

DWB is commonly prescribed to patients deemed to be at higher risk of complications and the role of selection bias in producing our results must be considered. Factors known to increase rates of complications include older age [25], smoking [26], alcohol [27], diabetes mellitus (DM) [28], obesity [29], OTA 42C fractures [30, 31] and open fracture [32]. Furthermore, it is possible that reamed nailing may minimise non-union [5, 33], while the effect of dynamic nailing on union time and complication rates has produced mixed results [34–36]. The influence of nail-to-canal diameter ratio on union rates has produced mixed results [31, 37], while the effect of the number of nails used has not been investigated. Studies utilised in meta-analysis for union time included one RCT [8]. The remaining three studies were all matched for age [6, 7, 23], two were matched for smoking and DM [7, 23] and one was matched for alcoholism [23]. All exclusively utilised reamed IMN except one which did not specify [7], and two exclusively utilised locked nailing [8, 23]. No studies commented on nail-to-canal diameter ratio and only one specified the number of nails used [8]. One study was matched for fracture type [6], and the remaining two excluded OTA 42C fractures. One study that could not be utilised for meta-analysis included 37 OTA 42C fractures and demonstrated equivocal union time between EWB and DWB groups [21]. Therefore, although unclear, our results indicate that EWB may produce faster union after locked, reamed IMN of OTA 42A, 42B, and possibly 42C, fractures in elderly, smoking, diabetic and alcoholic patients.

The two studies utilised in meta-analysis for complications included one matched for age [6], but none that matched for smoking, DM or obesity. One study matched for fracture type [6] and both excluded OTA 42C fractures. One study used reamed IMN [6], while the other did not specify. One study exclusively used locked nailing [22], while the other used both [6]. Two studies were not able to be included for meta-analysis as numbers of complications were not reported. These included an RCT of locked, reamed nailing [8] and one in which EWB and DWB groups were matched for age, smoking, obesity and fracture type [21]. The latter even included 37 OTA type 42C fractures. Both studies reported no significant increase in complications with EWB. Therefore, our results indicate that the role of EWB in at-risk patient groups is unclear, although preliminary randomised results report no increased risk of complications and matched results demonstrate no increased risk of complications in elderly, smoking and obese patients treated with locked, reamed IMN after OTA 42A-C fractures. Further investigation into the role of EWB in at-risk groups is certainly required and reporting of outcomes by OTA fracture type is encouraged.

The results of this study have significant potential advantages. As the most common long-bone fracture with an estimated cost of £8279 for surgical treatment [38], tibial shaft fractures confer a considerable burden to hospitals globally. Furthermore, the uses of intramedullary nailing for tibial shaft fractures are increasing. Historically, many tibial shaft fractures were treated with closed reduction and casting, as only unstable and open fractures necessitate surgical management [39]. However, IMN has become the gold standard as it has been associated with significantly decreased rates of malalignment, return to work and all SMFA domains at 3 months following IWB [40]. Furthermore, due to the subcutaneous location of the anteromedial tibia and frequent high-energy mechanisms, approximately 24% of tibial shaft fractures are open and require surgical management [1], with IMN shown to minimise re-operation rates [41]. As our study has identified that early post-operative weight-bearing was significantly correlated with earlier union time and reduced rates of complications, this approach may confer improved clinical outcomes to a large patient demographic and considerable cost savings to surgical systems globally.

Potential advantages of early weight-bearing after lower limb fractures have been recognised for many years. Garden first encouraged EWB after surgical fixation of femoral neck fractures in 1961 [42], and in 1979, da Costa and Kumar found that weight-bearing earlier than 6 weeks after conservative management of tibial shaft fractures produced faster union time and no increase in complications [43]. Since this time, evidence has encouraged IWB after surgical fixation of multiple lower limb fractures, including neck of femur fractures [42, 44], femoral shaft fractures [45, 46], ankle fractures [47–51] and Schatzker I–III plateau fractures [52–54]. IWB has also been advocated in tibial shaft fractures treated with external fixation or plate and screws [55, 56]. However, evidence comparing EWB and DWB after IMN of tibial shaft fractures is emerging rapidly, with three studies published on the topic in the last 2 years [7, 21, 22]. The emerging nature of this field along with the unclear role of EWB in at-risk groups demands a greater need for a formal synthesis of current evidence to update conceptions on the topic.

The results of our study need to be interpreted considering its strengths and weaknesses. The main limitation of this study is that patient groups between studies were not comparable and, therefore, the results of this study cannot be extended to the entire population. In particular, the outcomes of EWB were not reported for at-risk groups, preventing sub-group analysis. Second, there was a paucity of evidence as we were only able to include eight studies for analysis. Furthermore, the results of two studies were influenced by confounding factors [6, 7]. One included more butterfly-fragment fractures in the DWB group [7], while the other compared EWB and dynamic nailing with DWB and locked nails, possibly confounding results [6]. There was also a lack of standardised definitions of EWB and DWB. Four studies defined EWB by IWB [7, 8, 20, 23], while others used other time points. Similarly, two studies considered weight-bearing at 6 weeks to be DWB [7, 8], while others used later time points. There was also a lack of standardised functional outcomes measures, preventing meta-analysis. Future randomised or matched studies including and reporting outcomes for at-risk patient groups are required. Using standard time points for EWB and DWB, such as IWB post-operatively and weight-bearing at 6 weeks post-operatively or later would also be useful. Use of standardised functional and patient-reported outcome measure should also be encouraged.

## Conclusions

Based on the results of our study, EWB after IMN for tibial shaft fractures may produce faster union and fewer total complication in patients without clear risk factors. However, current evidence is minimal and has considerable limitations. In particular, the role of EWB in groups at higher risk of complications is yet to be determined. Further randomised studies reporting outcomes in these groups, with standardised definitions of comparators and measurements of patient-reported and functional outcomes are required.

## Supplementary Information

Below is the link to the electronic supplementary material.

Supplementary file1 (DOCX 152 KB)

## Data Availability

All data may be made available upon request.
