# Supplementary material for: Outcomes of early versus delayed weight-bearing with intramedullary nailing of tibial shaft fractures: a systematic review and meta-analysis
Source: Eur J Trauma Emerg Surg. 2022 Mar 3;48(5):3521–7. doi: 10.1007/s00068-022-01919-w (PMC9532312; doi:10.1007/s00068-022-01919-w)
Supplement: Supplementary file 1 — Supplementary file1 (DOCX 152 KB) [file 68_2022_1919_MOESM1_ESM.docx]

**SUPPLEMENTARY DIGITAL CONTENT**

**List of Supplementary Digital Content**

- Appendix 1: PRISMA 2020 Checklist
- Appendix 2: MOOSE Checklist
- Appendix 3: Prisma Flow Diagram detailing inclusion and exclusion of studies
- Appendix 4: Justification of exclusions at full-text review
- Appendix 5: Study Characteristics
- Appendix 6: Forest plot showing association of time to weight bearing after IM nailing of tibial shaft fractures with union time
- Appendix 7: Funnel plot showing publication bias for studies reporting on union time and association with time to weight bearing after IM nailing of tibial shaft fractures
- Appendix 8: Small-study effects for studies reporting on union time and association with time to weight bearing after IM nailing of tibial shaft fractures
- Appendix 9: Forest plot showing association of time to weight bearing after IM nailing of tibial shaft fractures with delayed union rate
- Appendix 10: Funnel plot showing publication bias for studies reporting on delayed union rate and association with time to weight bearing after IM nailing of tibial shaft fractures
- Appendix 11: Small-study effects for studies reporting on delayed union rate and association with time to weight bearing after IM nailing of tibial shaft fractures
- Appendix 12: Forest plot showing association of time to weight bearing after IM nailing of tibial shaft fractures with non-union rate
- Appendix 13: Funnel plot showing publication bias for studies reporting on non-union rate and association with time to weight bearing after IM nailing of tibial shaft fractures
- Appendix 14: Small-study effects for studies reporting on non-union rate and association with time to weight bearing after IM nailing of tibial shaft fractures
- Appendix 15: Forest plot showing association of time to weight bearing after IM nailing of tibial shaft fractures with malunion rate
- Appendix 16: Funnel plot showing publication bias for studies reporting on malunion rate and association with time to weight bearing after IM nailing of tibial shaft fractures
- Appendix 17: Small-study effects for studies reporting on malunion rate and association with time to weight bearing after IM nailing of tibial shaft fractures
- Appendix 18: Forest plot showing association of time to weight bearing after IM nailing of tibial shaft fractures with reoperation rate
- Appendix 19: Funnel plot showing publication bias for studies reporting on reoperation rate and association with time to weight bearing after IM nailing of tibial shaft fractures
- Appendix 20: Small-study effects for studies reporting on reoperation rate and association with time to weight bearing after IM nailing of tibial shaft fractures
- Appendix 21: Forest plot showing association of time to weight bearing after IM nailing of tibial shaft fractures with complication rate
- Appendix 22: Funnel plot showing publication bias for studies reporting on complication rate and association with time to weight bearing after IM nailing of tibial shaft fractures
- Appendix 23: Small-study effects for studies reporting on complication rate and association with time to weight bearing after IM nailing of tibial shaft fractures
- Appendix 24: Risk of bias scoring in observational studies
- Appendix 25: Risk of bias scoring in RCTs

**Appendix 1. PRISMA 2020 Checklist**

| **Section and Topic** | **Item #** | **Checklist item** | **Location where item is reported** |
| --- | --- | --- | --- |
| **TITLE** | | |  |
| Title | 1 | Identify the report as a systematic review. | Title page |
| **ABSTRACT** | | |  |
| Abstract | 2 | See the PRISMA 2020 for Abstracts checklist. | 3-4 |
| **INTRODUCTION** | | |  |
| Rationale | 3 | Describe the rationale for the review in the context of existing knowledge. | 5 |
| Objectives | 4 | Provide an explicit statement of the objective(s) or question(s) the review addresses. | 5 |
| **METHODS** | | |  |
| Eligibility criteria | 5 | Specify the inclusion and exclusion criteria for the review and how studies were grouped for the syntheses. | 6 |
| Information sources | 6 | Specify all databases, registers, websites, organisations, reference lists and other sources searched or consulted to identify studies. Specify the date when each source was last searched or consulted. | 6-7 |
| Search strategy | 7 | Present the full search strategies for all databases, registers and websites, including any filters and limits used. | 6-7, appendix |
| Selection process | 8 | Specify the methods used to decide whether a study met the inclusion criteria of the review, including how many reviewers screened each record and each report retrieved, whether they worked independently, and if applicable, details of automation tools used in the process. | 6-7 |
| Data collection process | 9 | Specify the methods used to collect data from reports, including how many reviewers collected data from each report, whether they worked independently, any processes for obtaining or confirming data from study investigators, and if applicable, details of automation tools used in the process. | 7 |
| Data items | 10a | List and define all outcomes for which data were sought. Specify whether all results that were compatible with each outcome domain in each study were sought (e.g. for all measures, time points, analyses), and if not, the methods used to decide which results to collect. | 6-7 |
|  | 10b | List and define all other variables for which data were sought (e.g. participant and intervention characteristics, funding sources). Describe any assumptions made about any missing or unclear information. | 6-7 |
| Study risk of bias assessment | 11 | Specify the methods used to assess risk of bias in the included studies, including details of the tool(s) used, how many reviewers assessed each study and whether they worked independently, and if applicable, details of automation tools used in the process. | 7 |
| Effect measures | 12 | Specify for each outcome the effect measure(s) (e.g. risk ratio, mean difference) used in the synthesis or presentation of results. | 7-8 |
| Synthesis methods | 13a | Describe the processes used to decide which studies were eligible for each synthesis (e.g. tabulating the study intervention characteristics and comparing against the planned groups for each synthesis (item #5)). | 7-8 |
|  | 13b | Describe any methods required to prepare the data for presentation or synthesis, such as handling of missing summary statistics, or data conversions. | 7-8 |
|  | 13c | Describe any methods used to tabulate or visually display results of individual studies and syntheses. | 7-8 |
|  | 13d | Describe any methods used to synthesize results and provide a rationale for the choice(s). If meta-analysis was performed, describe the model(s), method(s) to identify the presence and extent of statistical heterogeneity, and software package(s) used. | 7-8 |
|  | 13e | Describe any methods used to explore possible causes of heterogeneity among study results (e.g. subgroup analysis, meta-regression). | 7-8 |
|  | 13f | Describe any sensitivity analyses conducted to assess robustness of the synthesized results. | 7-8 |
| Reporting bias assessment | 14 | Describe any methods used to assess risk of bias due to missing results in a synthesis (arising from reporting biases). | 7-8 |
| Certainty assessment | 15 | Describe any methods used to assess certainty (or confidence) in the body of evidence for an outcome. | 7-8 |
| **RESULTS** | | |  |
| Study selection | 16a | Describe the results of the search and selection process, from the number of records identified in the search to the number of studies included in the review, ideally using a flow diagram. | 8 |
|  | 16b | Cite studies that might appear to meet the inclusion criteria, but which were excluded, and explain why they were excluded. | 8, appendix |
| Study characteristics | 17 | Cite each included study and present its characteristics. | 8, Table 1 |
| Risk of bias in studies | 18 | Present assessments of risk of bias for each included study. | 8, 10, Table 1, Table 3 |
| Results of individual studies | 19 | For all outcomes, present, for each study: (a) summary statistics for each group (where appropriate) and (b) an effect estimate and its precision (e.g. confidence/credible interval), ideally using structured tables or plots. | 8-11, figures 2-4, appendix |
| Results of syntheses | 20a | For each synthesis, briefly summarise the characteristics and risk of bias among contributing studies. | 8-11 |
|  | 20b | Present results of all statistical syntheses conducted. If meta-analysis was done, present for each the summary estimate and its precision (e.g. confidence/credible interval) and measures of statistical heterogeneity. If comparing groups, describe the direction of the effect. | 8-11, figures 2-4, appendix |
|  | 20c | Present results of all investigations of possible causes of heterogeneity among study results. | 8-11, figures 2-4, appendix |
|  | 20d | Present results of all sensitivity analyses conducted to assess the robustness of the synthesized results. | 8-11, figures 2-4, appendix |
| Reporting biases | 21 | Present assessments of risk of bias due to missing results (arising from reporting biases) for each synthesis assessed. | 8-11, figures, 2-4, appendix |
| Certainty of evidence | 22 | Present assessments of certainty (or confidence) in the body of evidence for each outcome assessed. | 8-11, figures 2-4, appendix |
| **DISCUSSION** | | |  |
| Discussion | 23a | Provide a general interpretation of the results in the context of other evidence. | 11 |
|  | 23b | Discuss any limitations of the evidence included in the review. | 13 |
|  | 23c | Discuss any limitations of the review processes used. | 13 |
|  | 23d | Discuss implications of the results for practice, policy, and future research. | 10-14 |
| **OTHER INFORMATION** | | |  |
| Registration and protocol | 24a | Provide registration information for the review, including register name and registration number, or state that the review was not registered. | 6 |
|  | 24b | Indicate where the review protocol can be accessed, or state that a protocol was not prepared. | 6 |
|  | 24c | Describe and explain any amendments to information provided at registration or in the protocol. | Not applicable |
| Support | 25 | Describe sources of financial or non-financial support for the review, and the role of the funders or sponsors in the review. | Title page |
| Competing interests | 26 | Declare any competing interests of review authors. | Title page |
| Availability of data, code and other materials | 27 | Report which of the following are publicly available and where they can be found: template data collection forms; data extracted from included studies; data used for all analyses; analytic code; any other materials used in the review. | Title page |

**Appendix 2. MOOSE Checklist**

| **Reporting Criteria** | **Reported (Yes/No)** | **Reported on Page Number** |
| --- | --- | --- |
| Problem definition | Yes | 5 |
| Hypothesis statement | Yes | 5 |
| Description of study outcomes | Yes | 6–7 |
| Type of exposure or intervention used | Yes | 6 |
| Type of study designs used | Yes | 6 |
| Study population | Yes | 6 |
| Qualifications of searchers (e.g. librarians and investigators) | No | - |
| Search strategy, including time period included in the synthesis and keywords | Yes | 6–7, Appendix |
| Effort to include all available studies, including contact with authors | Yes | 6–7 |
| Databases and registries searched | Yes | 6 |
| Search software used, name and version, including special features used (e.g. explosion) | No | - |
| Use of hand searching (e.g. reference lists of obtained articles) | No | - |
| List of citations located and those excluded, including justification | Yes | 8, Appendix |
| Methods of addressing articles published in languages other than English | Yes | 6 |
| Method of handling abstracts and unpublished studies | Yes | 6 |
| Description of any contact with authors | Yes | No contact with authors |
| Description of relevance or appropriateness of studies assembled for assessing the hypothesis to be tested | Yes | 6 |
| Rationale for the selection and coding of data (e.g. sound clinical principles or convenience) | Yes | 7–8 |
| Documentation of how data were classified and coded (e.g. multiple raters, blinding, and interrater reliability) | Yes | 7–8 |
| Assessment of confounding (e.g. comparability of cases and controls in studies where appropriate) | Yes | 7–8 |
| Assessment of study quality, including blinding of quality assessors; stratification or regression on possible predictors of study results | Yes | 7–8 |
| Assessment of heterogeneity | Yes | 7–9 |
| Description of statistical methods (e.g. complete description of fixed or random effects models, justification of whether the chosen models account for predictors of study results, dose-response models, or cumulative meta-analysis) in sufficient detail to be replicated | Yes | 7–8 |
| Provision of appropriate tables and graphics | Yes | Throughout manuscript |
| Graphic summarizing individual study estimates and overall estimate | Yes | Figures 2–4, appendix |
| Table giving descriptive information for each study included | Yes | Table 1 |
| Results of sensitivity testing (e.g. subgroup analysis) | Yes | 8–9 |
| Indication of statistical uncertainty of findings | Yes | 8–9 |
| Quantitative assessment of bias (e.g. publication bias) | Yes | 8–9 |
| Justification for exclusion (e.g. exclusion of non-English-language citations) | Yes | 8, Figure 1, Appendix |
| Assessment of quality of included studies | Yes | 11, Figure 1, Figure 3 |
| Consideration of alternative explanations for observed results | Yes | 11–13 |
| Generalization of the conclusions (i.e. appropriate for the data presented and within the domain of the literature review) | Yes | 11–14 |
| Guidelines for future research | Yes | 13–14 |
| Disclosure of funding source | Yes | Title page |

**Appendix 3: Prisma Flow Diagram detailing inclusion and exclusion of studies**

**
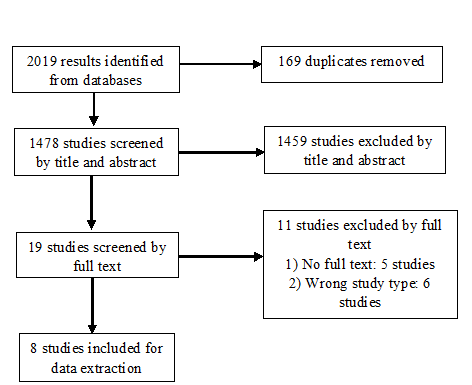
**

**Appendix 4. Justification of exclusions at full-text review**

**Studies excluded at full-text review**

**Wrong Outcomes**

(Outcomes not relevant or could not be extracted)

1. De Smet K, Mostert AK, De Witte J, De Brauwer V, Verdonk R. Closed intramedullary tibial nailing using the Marchetti-Vicenzi nail. *Injury.*; 31(8): 597–603

**Wrong Study Type**

(Non-observational study)

1. Kubiak EN, Beebe MJ, North K, Hitchcock R, Potter MQ. Early weight bearing after lower extremity fractures in adults. *J Am Acad Orthop Surg*.; 21(12): 727-38
2. Sanders R, Kellam J. Invited Commentary: To Weight-Bear or Not to Weight-Bear? Is That Really a Question?. *J Orthop Trauma*., 2016; 30(7): 375-6

**Wrong or No Comparator**

1. Franco-de la Torre L, Villafán‐Bernal JR, Garmendia‐Castañón R, Franco‐González AP, Isiordia‐Espinoza MA, Alcalá‐Zermeño JL, Gómez‐Sánchez E, Rodríguez‐Méndez LM, Sánchez‐Enríquez S. *Cirugia y cirujanos*, 2019; 87(1): 18‐22
2. Merianos P, Papagianna K, Scretas E, Smyrnis P. Ender nails for segmental tibial fracture. Early weight bearing in 22 cases. *Acta Orthop Scand.*; 59(3): 297-301

**Wrong Population**

1. Olerud S, Karlstrom G. The spectrum of intramedullary nailing of the tibia. *Clin Orthop Relat Res.,* 1986; (212): 101-12

**Full Text Unavaiable**

1. Bonnevialle P, Bellumore Y, Foucras L, Hézard L, Mansat M. [Tibial fracture with intact fibula treated by reamed nailing]. *Rev Chir Orthop Reparatrice Appar Mot*., 2000; 86(1): 29–37.
2. de La Caffiniere JY, Mignard JP, Le Balc’h T, Mazas F. [Early weight bearing after percutaneous nailing of the tibia in recent diaphyseal fractures of the leg (author’s transl)]. *Ann Chir*.; 31(6): 483-8
3. Latal, J. (2000) “Unreamed locking nailing of diaphyseal fractures”. *Lekarsky Obzor*., 2000; 49(9): 287-289
4. Tang C, Pang S, Wang N. The treatment of extremities fracture with intramedullary nail accomplished with postoperative functional exercises. *Chinese Journal of Clinical Rehabilitation*,; 7(2): 336
5. Tondelli P, Harder F. Postoperative treatment of osteosynthetic tibia shaft fractures. *Therapeutische Umschau*,; 37(9): 747-751

**Appendix 5: Study Characteristics**

| **Study** | **Apostolides et al., 2020** | **Bauwen et al., 2020** | **Greenhill et al., 2016** | **Gross et al., 2016** | **Hernandez-Vaquero et al., 2012** | **Houben et al., 2018** | **Schemitcsh et al., 2012** | **Uemi et al., 2020** |
| --- | --- | --- | --- | --- | --- | --- | --- | --- |
| **Country** | United Kingdom | France | United States of America | United States of America | Spain | Netherlands | Canada | Japan |
| **Setting** | Hospital | Hospital | Hospital | Hospital | Hospital | Hospital | Hospital | Hospital |
| **Study Design** | Retrospective cohort Study | Retrospective cohort Study | Retrospective cohort Study | Randomised Controlled Trial | Retrospective Case-Control Study | Retrospective cohort Study | Prospective Cohort Study | Prospective Cohort Study |
| **Follow-up** | 6-8 weeks | 1 year | 1 year | Until union (av 22 weeks) | 1 year | 1 year | 1 year | 1 year |
| **Fractures (n)** | 92 | 184 | 83 | 68 | 67 | 166 | 1226 | 263 |
| **Fracture type** | AO Fracture Types: 42A and 42B | AO Fracture Types: 42A and 42B, Gustillo I-III, | AO Fracture Type: 42A | AO Fracture Types: 42A-C | AO Fracture Types: 42A and 42B | AO Fracture Types: 42A-C | AO Fracture Types: 42A-C | AO Fracture Types: 42A-C |
| **Population (Intervention)** | Full weight-bearing immediately post-operatively | Weight-bearing before 45 days post-operatively | Weight-bear as tolerated immediately post-operatively | Weight-bear as tolerated immediately post-operatively | Dynamised nail and full weight-bearing before union, mostly before 4 weeks | Impaired Fracture Healing; no consolidation at 6 months | Full weight-bearing immediately post-operatively | Partial weight-bearing within 4 weeks post-operatively |
| **Population (Comparator)** | 1)No weight-bearing for 6 weeks 2)No weight-bearing for 2 weeks, then partial weight-bearing until 6 weeks postoperatively | Weight-bearing after 45 days post-operatively | No weight-bearing for 8 weeks | No weight-bearing for 6 weeks | Static nail and no weight-bearing until first sign of union | Normal fracture healing | 1)Partial weight-bearing 2)No weight-bearing (duration not clear) | No weight-bearing for at least 4 weeks post-operatively |
| **Source of Funding** | None | None | None | None | None | None | Research grants were received from the following: Canadian Institutes of Health Research (MCT-38140) | None |
| **Risk of Bias (%)** | 59.4 | 62.5 | 62.5 | **Low** | 59.4 | 56.3 | 59.4 | 56.3 |

**Appendix 6: Forest plot showing association of time to weight bearing after IM nailing of tibial shaft fractures with union time**

**Appendix 7: Funnel plot showing publication bias for studies reporting on union time and association with time to weight bearing after IM nailing of tibial shaft fractures**

**Appendix 8:** **Small-study effects for studies reporting on union time and association with time to weight bearing after IM nailing of tibial shaft fractures**

| Std Eff | Coef. | Std Err. | t | P>\|t\| | 95% Conf. Interval |
| --- | --- | --- | --- | --- | --- |
| Slope | -2.29 | 16.67 | -0.14 | 0.903 | -74.02, 69.43 |
| Bias | -0.05 | 6.92 | -0.01 | 0.995 | -29.83, 29.73 |

**Appendix 9: Forest plot showing association of time to weight bearing after IM nailing of tibial shaft fractures with delayed union rate**

**Appendix 10: Funnel plot showing publication bias for studies reporting on delayed union rate and association with time to weight bearing after IM nailing of tibial shaft fractures**

**Appendix 11: Small-study effects for studies reporting on delayed union rate and association with time to weight bearing after IM nailing of tibial shaft fractures**

| Std. Eff. | Coef. | Std. Err. | t | P>\|t\| | 95% Conf. Interval |
| --- | --- | --- | --- | --- | --- |
| Slope | -1.36 | 0.64 | -2.13 | 0.17 | -4.12, 1.39 |
| Bias | -2.45 | 1.02 | 2.40 | 0.14 | -1.94, 6.84 |

**Appendix 12:** **Forest plot showing association of time to weight bearing after IM nailing of tibial shaft fractures with non-union rate**

**Appendix 13:** **Funnel plot showing publication bias for studies reporting on non-union rate and association with time to weight bearing after IM nailing of tibial shaft fractures**

**Appendix 14: Small-study effects for studies reporting on non-union rate and association with time to weight bearing after IM nailing of tibial shaft fractures**

| Std. Eff. | Coef. | Std. Err. | t | P>\|t\| | 95% Conf. Interval |
| --- | --- | --- | --- | --- | --- |
| Slope | 0.49 | 0.74 | 0.66 | 0.55 | -1.58, 2.56 |
| Bias | -0.36 | 0.75 | -0.05 | 0.97 | -2.13, 2.06 |

**Appendix 15:** **Forest plot showing association of time to weight bearing after IM nailing of tibial shaft fractures with malunion rate**

**Appendix 16: Funnel plot showing publication bias for studies reporting on malunion rate and association with time to weight bearing after IM nailing of tibial shaft fractures**

**Appendix 17: Small-study effects for studies reporting on malunion rate and association with time to weight bearing after IM nailing of tibial shaft fractures**

| Std. Eff. | Coef. | Std. Err. | t | P>\|t\| | 95% Conf. Interval |
| --- | --- | --- | --- | --- | --- |
| Slope | 20.34 | - | - | - | - |
| Bias | -1.43 | - | - | - | - |

**Appendix 18: Forest plot showing association of time to weight bearing after IM nailing of tibial shaft fractures with reoperation rate**

**Appendix 19: Funnel plot showing publication bias for studies reporting on reoperation rate and association with time to weight bearing after IM nailing of tibial shaft fractures**

**Appendix 20: Small-study effects for studies reporting on reoperation rate and association with time to weight bearing after IM nailing of tibial shaft fractures**

| Std. Eff. | Coef. | Std. Err. | t | P>\|t\| | 95% Conf. Interval |
| --- | --- | --- | --- | --- | --- |
| Slope | 1.63 | - | - | - | - |
| Bias | -0.92 | - | - | - | - |

**Appendix 21:** **Forest plot showing association of time to weight bearing after IM nailing of tibial shaft fractures with complication rate**

**Appendix 22:** **Funnel plot showing publication bias for studies reporting on complication rate and association with time to weight bearing after IM nailing of tibial shaft fractures**

**Appendix 23: Small-study effects for studies reporting on complication rate and association with time to weight bearing after IM nailing of tibial shaft fractures**

| Std. Eff. | Coef. | Std. Err. | t | P>\|t\| | 95% Conf. Interval |
| --- | --- | --- | --- | --- | --- |
| Slope | 1.27 | - | - | - | - |
| Bias | -0.37 | - | - | - | - |

**Appendix 24:** **Risk of bias scoring in observational studies**

| Year | 2012 | 2018 | 2012 | 2020 | 2016 | 2020 | 2021 |
| --- | --- | --- | --- | --- | --- | --- | --- |
| Q1 Reviewer A | 1 | 1 | 1 | 1 | 1 | 1 | 1 |
| Q1 Reviewer B | 1 | 1 | 1 | 1 | 1 | 0 | 1 |
| Q1 Average A+B | 1 | 1 | 1 | 1 | 1 | 1 | 1 |
| Q2 Reviewer A | 1 | 1 | 1 | 1 | 1 | 1 | 1 |
| Q2 Reviewer B | 1 | 0.5 | 1 | 1 | 1 | 1 | 1 |
| Q2 Average A+B | 1 | 1 | 1 | 1 | 1 | 1 | 1 |
| Q3 Reviewer A | 1 | 1 | 1 | 1 | 1 | 1 | 1 |
| Q3 Reviewer B | 1 | 1 | 1 | 1 | 1 | 1 | 1 |
| Q3 Average A+B | 1 | 1 | 1 | 1 | 1 | 1 | 1 |
| Q4 Reviewer A | 1 | 1 | 1 | 1 | 1 | 1 | 1 |
| Q4 Reviewer B | 1 | 1 | 1 | 1 | 1 | 1 | 0 |
| Q4 Average A+B | 1 | 1 | 1 | 1 | 1 | 1 | 1 |
| Q5 Reviewer A (/2) | 0 | 2 | 1 | 2 | 2 | 2 | 1 |
| Q5 Reviewer B (/2) | 0 | 0 | 1 | 2 | 2 | 2 | 2 |
| Q5 Average A+B (/2) | 0 | 1 | 1 | 2 | 2 | 2 | 1 |
| Q6 Reviewer A | 1 | 1 | 1 | 1 | 1 | 1 | 1 |
| Q6 Reviewer B | 0 | 1 | 0 | 0 | 1 | 1 | 1 |
| Q6 Average A+B | 1 | 1 | 1 | 1 | 1 | 1 | 1 |
| Q7 Reviewer A | 1 | 1 | 1 | 1 | 1 | 1 | 1 |
| Q7 Reviewer B | 1 | 1 | 1 | 1 | 1 | 1 | 1 |
| Q7 Average A+B | 1 | 1 | 1 | 1 | 1 | 1 | 1 |
| Q8 Reviewer A | 1 | 1 | 0 | 0 | 0 | 0 | 0 |
| Q8 Reviewer B | 1 | 1 | 1 | 0 | 1 | 1 | 1 |
| 8 Average A+B | 1 | 1 | 1 | 0 | 0 | 0 | 0 |
| Q9 Reviewer A | 1 | 0 | 1 | 0 | 1 | 1 | 1 |
| Q9 Reviewer B | 1 | 1 | 1 | 1 | 1 | 1 | 1 |
| Q9 Average A+B | 1 | 0 | 1 | 0 | 1 | 1 | 1 |
| Q10 Reviewer A | 1 | 1 | 1 | 1 | 1 | 1 | 1 |
| Q10 Reviewer B | 1 | 1 | 1 | 1 | 1 | 1 | 1 |
| Q10 Average A+B | 1 | 1 | 1 | 1 | 1 | 1 | 1 |
| REPORTING Reviewer A (/11) | 9 | 10 | 9 | 9 | 10 | 10 | 9 |
| REPORTING Reviewer B (/11) | 8 | 8.5 | 9 | 9 | 11 | 10 | 10 |
| REPORTING Average A+B (/11) | 9 | 9 | 10 | 9 | 10 | 10 | 9 |
| Q11 Reviewer A | 1 | 0 | 1 | 1 | 1 | 1 | 0 |
| Q11 Reviewer B | 1 | 1 | 1 | 1 | 1 | 1 | 1 |
| Q11 Average A+B | 1 | 0 | 1 | 1 | 1 | 1 | 1 |
| Q12 Reviewer A | 0 | 1 | 1 | 1 | 1 | 1 | 1 |
| Q12 Reviewer B | 1 | 1 | 1 | 1 | 1 | 1 | 1 |
| Q12 Average A+B | 0 | 1 | 1 | 1 | 1 | 1 | 1 |
| Q13 Reviewer A | 1 | 0 | 1 | 0 | 0 | 0 | 0 |
| Q13 Reviewer B | 1 | 1 | 1 | 1 | 1 | 1 | 1 |
| Q13 Average A+B | 1 | 0 | 1 | 0 | 0 | 0 | 1 |
| EXTERNAL VALIDITY Reviewer A (/3) | 2 | 1 | 3 | 2 | 2 | 2 | 1 |
| EXTERNAL VALIDITY Reviewer B (/3) | 3 | 3 | 3 | 3 | 3 | 3 | 3 |
| EXTERNAL VALIDITY Average A+B (/3) | 2 | 1 | 3 | 2 | 2 | 2 | 3 |
| Q14 Reviewer A | 0 | 0 | 0 | 0 | 0 | 0 | 0 |
| Q14 Reviewer B | 0 | 0 | 0 | 0 | 0 | 0 | 0 |
| Q14 Average A+B | 0 | 0 | 0 | 0 | 0 | 0 | 0 |
| Q15 Reviewer A | 0 | 0 | 0 | 0 | 0 | 0 | 0 |
| Q15 Reviewer B | 1 | 1 | 1 | 0.5 | 1 | 0.5 | 1 |
| Q15 Average A+B | 0 | 0 | 0 | 0 | 0 | 0 | 0 |
| Q16 Reviewer A | 1 | 1 | 0 | 1 | 1 | 1 | 1 |
| Q16 Reviewer B | 0 | 0.5 | 0 | 0 | 0.5 | 0.5 | 0 |
| Q16 Average A+B | 1 | 1 | 0 | 1 | 1 | 1 | 1 |
| Q17 Reviewer A | 1 | 1 | 1 | 1 | 1 | 1 | 1 |
| Q17 Reviewer B | 1 | 1 | 1 | 1 | 1 | 1 | 1 |
| Q17 Average A+B | 1 | 1 | 1 | 1 | 1 | 1 | 1 |
| Q18 Reviewer A | 1 | 1 | 1 | 1 | 1 | 1 | 1 |
| Q18 Reviewer B | 1 | 1 | 1 | 1 | 1 | 1 | 1 |
| Q18 Average A+B | 1 | 1 | 1 | 1 | 1 | 1 | 1 |
| Q19 Reviewer A | 1 | 1 | 1 | 1 | 1 | 1 | 1 |
| Q19 Reviewer B | 0 | 1 | 1 | 1 | 1 | 1 | 1 |
| Q19 Average A+B | 1 | 1 | 1 | 1 | 1 | 1 | 1 |
| Q20 Reviewer A | 1 | 1 | 1 | 1 | 1 | 1 | 1 |
| Q20 Reviewer B | 1 | 1 | 1 | 1 | 1 | 1 | 1 |
| Q20 Average A+B | 1 | 1 | 1 | 1 | 1 | 1 | 1 |
| BIAS Reviewer A (/7) | 5 | 5 | 4 | 5 | 5 | 5 | 5 |
| BIAS Reviewer B (/7) | 4 | 5.5 | 5 | 4.5 | 5.5 | 5 | 5 |
| BIAS Average A+B (/7) | 5 | 5 | 4 | 5 | 5 | 5 | 5 |
| Q21 Reviewer A | 1 | 1 | 1 | 1 | 1 | 1 | 1 |
| Q21 Reviewer B | 1 | 1 | 0 | 1 | 1 | 1 | 1 |
| Q21 Average A+B | 1 | 1 | 1 | 1 | 1 | 1 | 1 |
| Q22 Reviewer A | 1 | 1 | 1 | 1 | 1 | 0 | 1 |
| Q22 Reviewer B | 1 | 1 | 1 | 0 | 1 | 0 | 1 |
| Q22 Average A+B | 1 | 1 | 1 | 0 | 1 | 0 | 1 |
| Q23 Reviewer A | 1 | 0 | 1 | 0 | 1 | 0 | 0 |
| Q23 Reviewer B | 0 | 0 | 0 | 0 | 0 | 0 | 0 |
| Q23 Average A+B | 0 | 0 | 0 | 0 | 0 | 0 | 0 |
| Q24 Reviewer A | 0 | 0 | 0 | 0 | 0 | 0 | 0 |
| Q24 Reviewer B | 0 | 0 | 0 | 0 | 0 | 0 | 0 |
| Q24 Average A+B | 0 | 0 | 0 | 0 | 0 | 0 | 0 |
| Q25 Reviewer A | 0 | 1 | 0 | 1 | 1 | 1 | 0 |
| Q25 Reviewer B | 0 | 0 | 0 | 0 | 0 | 0 | 0 |
| Q25 Average A+B | 0 | 0 | 0 | 0 | 0 | 0 | 0 |
| Q26 Reviewer A | 1 | 0 | 0 | 1 | 1 | 1 | 1 |
| Q26 Reviewer B | 1 | 1 | 1 | 0 | 1 | 1 | 1 |
| Q26 Average A+B | 1 | 1 | 0 | 1 | 1 | 1 | 1 |
| CONFOUNDING Reviewer A (/6) | 4 | 3 | 3 | 4 | 5 | 3 | 3 |
| CONFOUNDING Reviewer B (/6) | 3 | 3 | 2 | 1 | 3 | 2 | 3 |
| CONFOUNDING Average A+B (/6) | 3 | 3 | 2 | 2 | 3 | 2 | 3 |
| POWER Reviewer A (/5) | 0 | 0 | 0 | 0 | 0 | 0 | 0 |
| POWER Reviewer B (/5) | 0 | 0 | 0 | 0 | 0 | 0 | 0 |
| POWER Average A+B (/5) | 0 | 0 | 0 | 0 | 0 | 0 | 0 |
| TOTAL Reviewer A (/32) | 20 | 19 | 19 | 20 | 22 | 20 | 18 |
| TOTAL Reviewer B (/32) | 18 | 20 | 19 | 17.5 | 22.5 | 20 | 21 |
| TOTAL Average A+B (/32) | 19 | 18 | 19 | 18 | 20 | 19 | 20 |

**Appendix 25: Risk of bias scoring in RCTs**

**Domain 1: Risk of bias arising from the randomization process**

| **1.1 Was the allocation sequence random?** | Y / PY / PN / N / NI |
| --- | --- |
| **1.2 Was the allocation sequence concealed until participants were enrolled and assigned to interventions?** | Y / PY / PN / N / NI |
| **1.3 Did baseline differences between intervention groups suggest a problem with the randomization process?** | Y / PY / PN / N / NI |
| **Risk-of-bias judgement** | Low / High / Some concerns |

Domain 2: Risk of bias due to deviations from the intended interventions (effect of assignment to intervention)

| **2.1. Were participants aware of their assigned intervention during the trial?** | Y / PY / PN / N / NI |
| --- | --- |
| **2.2. Were carers and people delivering the interventions aware of participants' assigned intervention during the trial?** | Y / PY / PN / N / NI |
| **2.3. If Y/PY/NI to 2.1 or 2.2: Were there deviations from the intended intervention that arose because of the trial context?** | NA / Y / PY / PN / N / NI |
| **2.4 If Y/PY to 2.3: Were these deviations likely to have affected the outcome?** | NA / Y / PY / PN / N / NI |
| **2.5. If Y/PY/NI to 2.4: Were these deviations from intended intervention balanced between groups?** | NA / Y / PY / PN / N / NI |
| **2.6 Was an appropriate analysis used to estimate the effect of assignment to intervention?** | Y / PY / PN / N / NI |
| **2.7 If N/PN/NI to 2.6: Was there potential for a substantial impact (on the result) of the failure to analyse participants in the group to which they were randomized?** | NA / Y / PY / PN / N / NI |
| **Risk-of-bias judgement** | Low / High / Some concerns |

Domain 3: Missing outcome data

| **3.1 Were data for this outcome available for all, or nearly all, participants randomized?** | Y / PY / PN / N / NI |
| --- | --- |
| **3.2 If N/PN/NI to 3.1: Is there evidence that the result was not biased by missing outcome data?** | NA / Y / PY / PN / N |
| **3.3 If N/PN to 3.2: Could missingness in the outcome depend on its true value?** | NA / Y / PY / PN / N / NI |
| **3.4 If Y/PY/NI to 3.3: Is it likely that missingness in the outcome depended on its true value?** | NA / Y / PY / PN / N / NI |
| **Risk-of-bias judgement** | Low / High / Some concerns |

Domain 4: Risk of bias in measurement of the outcome

| **4.1 Was the method of measuring the outcome inappropriate?** | Y / PY / PN / N / NI |
| --- | --- |
| **4.2 Could measurement or ascertainment of the outcome have differed between intervention groups?** | Y / PY / PN / N / NI |
| **4.3 If N/PN/NI to 4.1 and 4.2: Were outcome assessors aware of the intervention received by study participants?** | NA / Y / PY / PN / N / NI |
| **4.4 If Y/PY/NI to 4.3: Could assessment of the outcome have been influenced by knowledge of intervention received?** | NA / Y / PY / PN / N / NI |
| **4.5 If Y/PY/NI to 4.4:** **Is it likely that assessment of the outcome was influenced by knowledge of intervention received?** | NA / Y / PY / PN / N / NI |
| **Risk-of-bias judgement** | Low / High / Some concerns |

Domain 5: Risk of bias in selection of the reported result

| **5.1 Were the data that produced this result analysed in accordance with a pre-specified analysis plan that was finalized before unblinded outcome data were available for analysis?** | Y / PY / PN / N / NI |
| --- | --- |
| **5.2. Is the numerical result being assessed likely to have been selected, on the basis of the results, from multiple eligible outcome measurements (e.g. scales, definitions, time points) within the outcome domain?** | Y / PY / PN / N / NI |
| **5.3 Is the numerical result being assessed likely to have been selected, on the basis of the results, from multiple eligible analyses of the data?** | Y / PY / PN / N / NI |
| **Risk-of-bias judgement** | Low / High / Some concerns |

Overall risk of bias

| **Risk-of-bias judgement** | Low / High / Some concerns |
| --- | --- |
